# Exposure to household pesticides and Parkinson's disease in the Parkinson's Progression Markers Initiative cohort

**DOI:** 10.3389/fneur.2024.1411468

**Published:** 2024-08-12

**Authors:** Bruno Lopes Santos-Lobato, Artur Francisco S. Schuh

**Affiliations:** ^1^Laboratório de Neuropatologia Experimental, Universidade Federal do Pará, Belém, Brazil; ^2^Hospital Ophir Loyola, Belém, Brazil; ^3^Serviço de Neurologia, Hospital de Clínicas de Porto Alegre, Porto Alegre, Brazil; ^4^Departamento de Farmacologia, Universidade Federal do Rio Grande do Sul, Porto Alegre, Brazil

**Keywords:** pesticide, Parkinson's disease, risk, cognition, Parkinson's Progression Markers Initiative

## Abstract

**Background:**

In the last decades, the association of household pesticide usage with Parkinson's disease (PD) has been poorly explored, with discordant results. Based on the Parkinson's Progression Markers Initiative (PPMI) cohort study, we analyzed (1) the association of household pesticide exposure with the development of PD and (2) the effect of household pesticides on progression of PD.

**Methods:**

Data from participants of the “FOllow Up persons with Neurologic Disease” (FOUND study) included in the PPMI cohort database were analyzed. The PPMI FOUND study applied the Parkinson's Disease Risk Factor Questionnaire to collect information regarding the use of pesticides in non-work settings during periods of life, and the lifetime pesticide exposure for each participant was estimated. We defined a high use of pesticides if the exposure estimate had a z-score higher than one standard deviation from the mean. Also, we evaluated longitudinal data of people with PD to analyze the effect of high use of household pesticides on disease progression according to motor impairment, cognitive dysfunction, depressive symptoms, and modification of motor clinical phenotype.

**Results:**

We analyzed data from 206 people with PD and 64 healthy controls, almost all from the USA. High use of household pesticides was not associated with the odds of developing PD. Regarding PD progression, only cognitive dysfunction was associated with the high use of household fungicides (HR 5.64 per standard deviation increase in exposure estimate, 95% CI 1.41–22.6).

**Conclusions:**

Chronic exposure to household pesticides may impact the clinical progression of PD, especially cognitive symptoms.

## 1 Introduction

Parkinson's disease (PD) is the neurological disease with the fastest growth in prevalence worldwide (1). The increasing number of PD cases may be partially attributed to environmental factors, such as pesticide exposure (2). Global pesticide commercialization for occupational (mainly in agriculture) and household settings has increased in the last decades (3), and the current climate changes may potentialize their use to expand food production and control disease vectors (4).

Household pesticides are pest control products for use in and around homes and gardens, including insecticides, herbicides, and fungicides. Household pesticides are commercialized in many forms, such as sprays, baits, heated liquid dispensers, mosquito coils, and smoke generators. Many contain substances causing oxidative damage and mitochondrial dysfunction, and some were demonstrated to induce nigrostriatal dopaminergic damage in animal models (5). Their use is common in high-income countries as well as in low- and middle-income countries. However, the informal market and the incorrect use of these products result in a higher risk of intoxication for vulnerable communities (6).

Despite the growing population exposure to household pesticides, few studies have explored the association between these products (7–10). Based on data from the Parkinson's Progression Markers Initiative (PPMI) cohort study, we analyzed the association of household pesticide exposure with the development of PD and its progression.

## 2 Materials and methods

### 2.1 Study design

This study used data from the PPMI cohort (http://www.ppmi-info.org/data). The PPMI is an ongoing longitudinal multicentric cohort study to identify biomarkers of PD progression, which was designed as a case-control study with accompanying prospective follow-up of people with PD (11). We conducted two analyses: (I) association between exposure to specific household pesticide groups and PD (case-control design), and (II) association between exposure to specific household pesticide groups and outcomes of PD progression (longitudinal design based on prospective follow-up). Data used in this study were obtained from the PPMI database on September 1, 2023. We selected only participants from PPMI who were also enrolled in the “FOllow Up persons with Neurologic Disease” (FOUND study).

The PPMI FOUND participants were evaluated at baseline for collecting health and life-long lifestyle information through the Parkinson's Disease Risk Factor Questionnaire (PDRFQ) (12). The participants were divided into two groups: people with PD and healthy controls (people with no neurologic disorder and no first-degree relative with PD). Individuals with scans without evidence of dopaminergic deficit (SWEDD) and atypical forms of parkinsonism were excluded.

### 2.2 Household pesticide use

We extracted data from the PDRFQ for the use of pesticides in non-work settings, which stratifies the usage according to the pesticide group (insecticides, fungicides, herbicides) and the period of life with exposure (birth to age 25, age 26–35, age 36–45, age 46–55, age 56–65, age 66 and above). Participants were asked if they used insecticides to kill bugs such as ants, roaches, mites, or other pests (including any used on pets); fungicides to kill mold, mildew, or rot; herbicides to kill weeds or plants in or around their home, lawn, or garden during specific periods of time. If yes, they were asked how often the pesticide groups were used during specific periods of time (three categories of answers: rarely−1–2 times/year, occasionally−3–6 times/year, often—more than six times/year).

To estimate the lifelong household use for any pesticide group, the categories of answers were transformed into numbers (1—rarely, 2—occasionally, 3—often), and the values were summed for all evaluated periods of life (exposure estimate). After, we log- and z-transformed the exposure estimates for each pesticide group to ensure normalization. We defined a high use of specific household pesticide groups if the exposure estimate had a *z*-score higher than 1 standard deviation from the mean. We also extracted the following variables: sex, residential history, age at evaluation, age at PD onset (for people with PD), and lifetime smoking history.

### 2.3 Outcomes of progression of Parkinson's disease

For disease progression, we selected the Movement Disorders Society Unified Parkinson's Disease Rating Scale Part 3 (MDS-UPDRS 3) to assess motor function (13); the Montreal Cognitive Assessment (MoCA) to assess cognitive status (14); the 15-item Geriatric Depression Scale (GDS) to assess depressive symptoms (15); and the tremor-dominant (TD) and the postural instability and gait disturbance (PIGD) scores derived from MDS-UPDRS to assess the motor phenotype status (16). We extracted data from each outcome's annual evaluation from the baseline to the last registered year.

For the time-to-event analysis, we defined as an event: high motor impairment (MDS-UPDRS 3 ≥35), high cognitive dysfunction (MoCA ≤ 20), high levels of depressive symptoms (GDS ≥5), and conversion from the TD to the PIGD motor phenotype. The time difference was the years between the baseline evaluation and the first follow-up evaluation date in which the event was present (10).

### 2.4 Statistical analysis

#### 2.4.1 Association between exposure to specific household pesticide groups and Parkinson's disease

For the association between the use of specific (insecticides, fungicides, herbicides) and overall household pesticides and diagnosis of PD, we performed multivariate logistic regression with a diagnosis of PD (PD vs. healthy controls) as the dependent variable. Odds ratios (OR) were estimated. Independent variables were sex, years of education, age at evaluation, residential history (people born and living in the USA vs. migrants), lifetime smoking history (if participants smoked at least 100 cigarettes—about five packs—in their entire lifetime), high use of household pesticide groups. High use of specific and overall household pesticides was added to the models as a categorical variable (yes/no).

#### 2.4.2 Association between exposure to specific household pesticide groups and outcomes of Parkinson's disease progression

To analyze the effect of household pesticides on four outcomes of PD progression (MDS-UPDRS 3 ≥35, MoCA ≤ 20, GDS ≥5, conversion from TD to PIGD motor phenotype), we performed a Cox proportional hazards model with the follow-up time starting from the baseline evaluation. Hazard ratios (HR) were estimated. Independent variables included were sex, age at disease onset, years of education, levodopa equivalent daily doses at baseline, lifetime smoking history, and high use of household pesticide groups.

Missing data were excluded. All analyses were performed using SPSS for Windows version 23.0 (SPSS Inc., Chicago, USA).

## 3 Results

### 3.1 Clinical characteristics of the sample

We analyzed a total of 363 participants. We excluded 93 participants (73 people with PD and 20 healthy controls) due to missing data about the use of household pesticides. A total of 270 participants (206 people with PD and 64 healthy controls) were eligible for analysis (Table 1). All 206 people with PD were followed up for at least 2 years after baseline evaluation and by a median of 7 years. Between people with PD and controls, there was no difference in sex proportion, smoking, use of specific household pesticide groups per year, and high exposure to specific household pesticide groups. Almost all participants were born and living in the USA.

**Table 1:** Clinical characteristics of the sample.

**Variable**	**People with PD (*n* = 206)**	**Healthy controls (*n* = 64)**	***P*-value**
Male sex, % (*n*)	59.2 (122)	67.2 (43)	0.30
Age at baseline^a^	61.6 (55–68)	59.4 (56–68)	0.78
Age at onset^a^	60.4 (53–67)	NA	NA
Less than 1 year of disease duration at baseline, % (*n*)	66 (136)	NA	NA
Years of education^a^	16 (16–19)	16 (16–18)	0.96
Born and living in the USA, % (*n*)	87.8 (180)	92.2 (59)	0.49
Early-onset PD, % (*n*)	16 (33)	NA	NA
Lifetime smoking history^b^, % (*n*)	32 (65)	45.3 (29)	0.07
Not using antiparkinsonian drugs at baseline, % (*n*)	70.6 (144)	NA	NA
Baseline MDS-UPDRS Part 3^a^	20 (15–25)	NA	NA
Depressive symptoms at baseline^c^, % (*n*)	13.7 (28)	NA	NA
High cognitive dysfunction at baseline^d^, % (*n*)	0 (0)	NA	NA
Tremor-dominant motor phenotype at baseline, % (*n*)	67.3 (134)	NA	NA
Lifetime use of household insecticides^e^, % (*n*)	96.6 (199)	95.3 (61)	0.70
Lifetime use of household fungicides, % (*n*)	28.2 (58)	29.7 (19)	0.87
Lifetime use of household herbicides, % (*n*)	80.1 (165)	82.8 (53)	0.71
Lifetime use of any household pesticides, % (*n*)	97.1 (200)	96.6 (62)	1.00
High use of household insecticides^f^, % (*n*)	25.7 (53)	25 (16)	1.00
High use of household fungicides, % (*n*)	4.9 (10)	1.6 (1)	0.53
High use of household herbicides, % (*n*)	43.2 (89)	46.9 (30)	0.45
High use of any household pesticides, % (*n*)	36.4 (75)	35.9 (23)	0.44

NA, not applied; PD, Parkinson's disease.

^a^Values in median (interquartile range).

^b^Lifetime smoking history (categorized as yes/no) was defined if participant smoked at least 100 cigarettes (about 5 packs) in entire lifetime.

^c^Depressive symptoms were defined if the Geriatric Depressive Score ≥ 5.

^d^High cognitive dysfunction was defined if the Montreal Cognitive Assessment ≤ 20.

^e^Lifelong use of household pesticide groups (categorized as yes/no) was defined if participants described the use of the specific pesticide in entire lifetime.

^f^High use of household pesticide groups (categorized as yes/no) was defined if the exposure estimate had a z-score higher than 1 standard deviation from the mean, and the exposure estimate was estimated by transforming the categories of answers into numbers and summing the values for all evaluated periods of life.

Most people with PD had < 1 year of disease onset and used no antiparkinsonian drugs at baseline evaluation. About two-thirds of these people had a TD motor phenotype, and most people with PD had mild motor scores at baseline (17). Less than 15% of people with PD had depressive symptoms, and no participant was diagnosed with cognitive dysfunction.

### 3.2 Association between exposure to specific household pesticide groups and Parkinson's disease

The multivariate logistic regression showed no association between the high use of specific and overall household pesticides and the odds of developing PD (Table 2).

**Table 2:** Association between household pesticide exposure and Parkinson's disease.

**Clinical variables**	***n* PD**	***n* CT**	**B**	**aOR**	**95% CI**	***P*-value**
Male sex	–	–	−0.347	0.70	0.38–1.30	0.26
Age at evaluation	–	–	0.009	1.00	0.97–1.04	0.59
Lifetime smoking history^*^	–	–	−0.564	0.56	0.31–1.03	0.06
Born and living in the USA	–	–	−0.598	0.55	0.19–1.53	0.25
Education	–	–	0.013	1.01	0.90–1.13	0.81
High use of household insecticides^**^	53	16	0.360	1.43	0.61–3.33	0.40
High use of household fungicides^**^	10	1	0.615	1.85	0.38–8.83	0.44
High use of household herbicides^**^	89	30	0.285	1.33	0.59–2.98	0.49
High use of any household pesticides^**^	75	23	0.360	1.43	0.61–3.33	0.40

B, regression coefficient; aOR, adjusted odds ratio; 95% CI, 95% confidence interval; n CT, number of control individuals from high pesticide exposure groups included in the multivariate model; n PD, number of people with Parkinson's disease from high pesticide exposure groups included in the multivariate model.

^*^Lifetime smoking history (categorized as yes/no) was defined if participant smoked at least 100 cigarettes (about 5 packs) in entire lifetime.

^**^High use of household pesticide groups (categorized as yes/no) was defined if the exposure estimate had a z-score higher than 1 standard deviation from the mean, and the exposure estimate was estimated by transforming the categories of answers into numbers and summing the values for all evaluated periods of life.

### 3.3 Association between exposure to specific household pesticide groups and outcomes of Parkinson's disease progression

**Figure 1:**
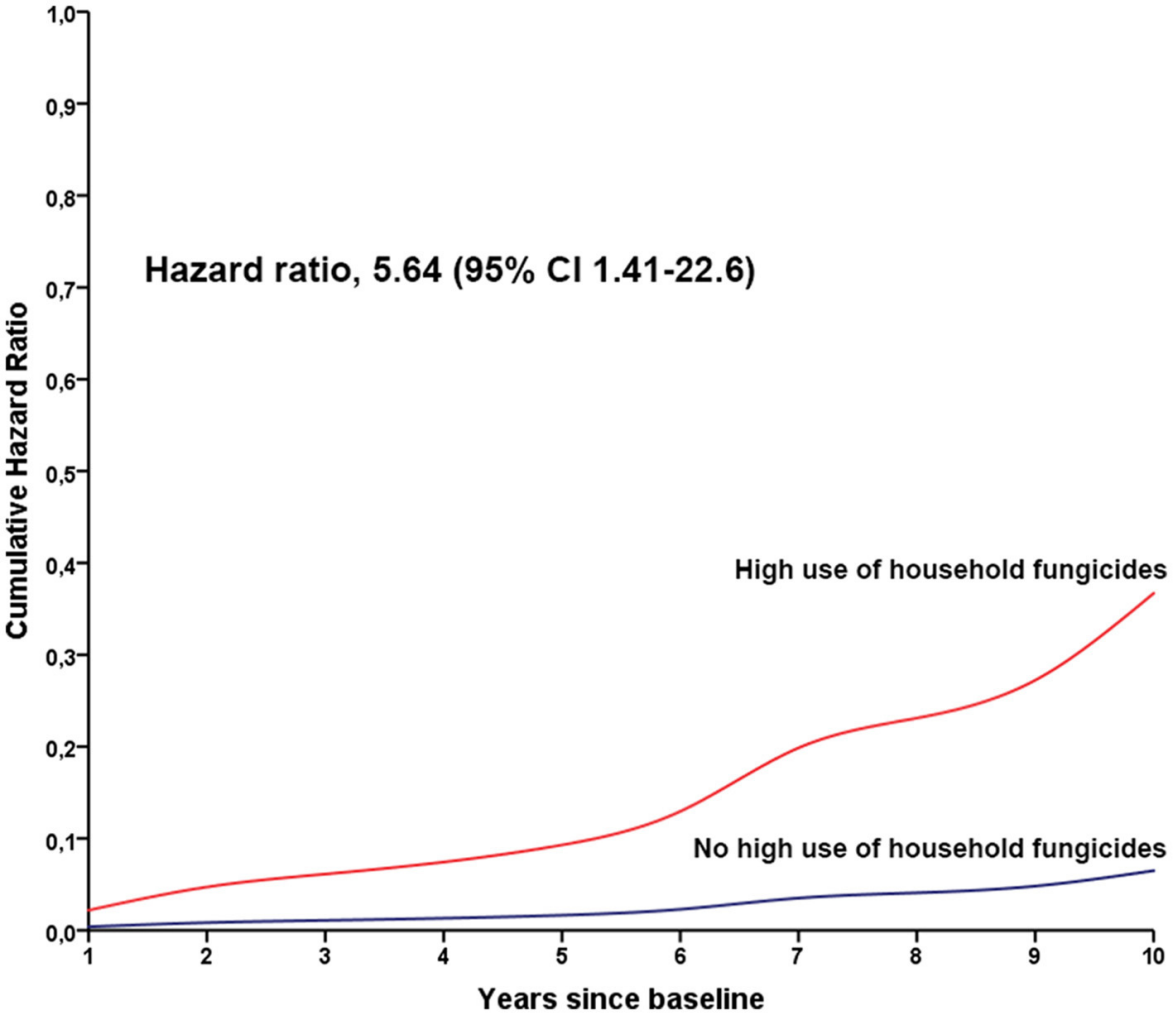
Cumulative hazard ratios for cognitive decline in people with Parkinson's disease according to the high use of household fungicides. The red line indicates the annual hazard ratio for people with high use of household fungicides, and the blue line indicates the annual hazard ratio for people without high use of household fungicides.

No associations were found between exposure to specific household pesticide groups and motor function decline, depression, and conversion from TD to the PIGD motor phenotype (Table 3). However, cognitive dysfunction (MoCA < 20) occurred faster in people with high use of household fungicides (HR 5.64 per standard deviation increase in exposure estimate, 95% CI 1.41–22.6; Table 3; Figure 1).

**Table 3:** Hazard ratios for outcomes of Parkinson's disease progression, according to household pesticide exposure.

**Outcomes of PD progression**	***n* PD**	***n* Events in PD**	**B**	**HR**	**95% CI**	***P*-value**
**MDS-UPDRS Part 3** ≥**35**						
High use of household insecticides	53	18	0.095	1.10	0.65–1.84	0.71
High use of household fungicides	10	3	0.905	2.47	1.13–5.38	0.02^*^
High use of household herbicides	89	11	0.344	1.41	0.81–2.45	0.22
**Geriatric Depression Scale** ≥**5**						
High use of household insecticides	53	10	−0.036	0.96	0.49–1.88	0.91
High use of household fungicides	10	1	0.208	1.23	0.42–3.57	0.70
High use of household herbicides	89	6	0.384	1.46	0.75–2.85	0.25
**Montreal Cognitive Assessment** ≤ **20**						
High use of household insecticides	53	4	−0.868	0.42	0.10–1.68	0.22
High use of household fungicides	10	1	1.731	5.64	1.41–22.6	**0.01**
High use of household herbicides	89	3	−0.131	0.87	0.23–3.35	0.84
**Conversion from TD to PIGD**						
High use of household insecticides	53	21	−0.072	0.93	0.58–1.47	0.76
High use of household fungicides	10	3	−0.421	0.65	0.26–1.62	0.36
High use of household herbicides	89	11	−0.070	0.93	0.57–1.52	0.77

High use of household pesticide groups (categorized as yes/no) was defined if the exposure estimate had a z-score higher than 1 standard deviation from the mean, and the exposure estimate was estimated by transforming the categories of answers into numbers and summing the values for all evaluated periods of life. Bold text indicates statistically significant associations.

PD, Parkinson's disease; B, Cox regression coefficient; HR, hazard ratio; 95% CI, 95% confidence interval; MDS-UPDRS, Movement Disorders Society Unified Parkinson's Disease Rating Scale; n PD, number of people with Parkinson's disease from high pesticide exposure groups included in the multivariate model; n Events in PD, number of events (MDS-UPDRS Part 3 ≥ 35, Geriatric Depression Scale ≥ 5, Montreal Cognitive Assessment ≤ 20, conversion from TD to PIGD) occurred with people with Parkinson's disease during the period of follow-up; TD, tremor-dominant motor phenotype; PIGD, postural instability and gait disturbance motor phenotype.

^*^Despite an assumed association between the risk of motor function decline and high use of household fungicides (represented by positive hazard ratio and 95% confidence intervals and P-value < 0.05), the Cox regression full model (including all independent variables) did not have a statistical significance according to the likelihood ratio test of the fit relative to a null (−2 ^*^ log-likelihood = 745; likelihood ratio chi-square statistics = 9.31, P-value = 0.156). Thus, the present analysis cannot confirm the association between the risk of motor function decline and the high use of household fungicides.

## 4 Discussion

Our results did not show an association between the high use of household pesticides and the onset of PD in a US cohort. Regarding the association between the high use of household pesticides and outcomes of PD progression, people with PD and high use of household fungicides have five times the probability of developing cognitive dysfunction, independent of sex, age, antiparkinsonian drug doses, smoking, and education.

Almost all participants had a history of lifetime use of household pesticides, mainly for insecticides and herbicides, with the highest proportion of household pesticide use described. Previous studies showed that between 50 and 70% of people in different countries use household pesticides (7, 9).

Clinical evidence regarding household pesticide exposure and PD is scarce. A previous study in the USA (California) showed that frequent household pesticide use increased the odds of developing PD (adjusted OR 1.47, 95% CI 1.13–1.92), with a relevant contribution of organophosphates (8). More recently, a Brazilian cohort showed that people exposed to household pesticides more than 30 days per year had a higher risk of developing PD (adjusted OR 2.27, 95% CI 1.46–3.52) (9). However, a population-based study in the USA (Washington State) did not show an effect on household pesticide exposure and the onset of PD (7).

The variability of results in these studies may be associated with the heterogeneity of methods used to evaluate household pesticide exposure (Supplementary Table 1). For example, the categories of answers regarding the frequency of pesticide use were different for each study; while some authors defined a high frequency of use as over 12 days per year (8, 9), the questionnaire used in the present study defined as over 6 days per year. Considering that household pesticide exposure is included in logistic regression models as a categorical variable, the variability of a high-tier frequency exposure between studies impairs their comparability.

Regarding the association between the high use of fungicides and faster cognitive decline, a recent study in the USA (California) reported that copper sulfate (pentahydrate), a common fungicide, was associated with motor and non-motor symptoms worsening, including faster high cognitive dysfunction in occupational and household settings (10). Furthermore, the relationship between parkinsonism and another fungicide, maneb, is well-recognized (18). Also, the dream-enacting behavior described in the synucleinopathy-related REM behavior disorder occurred more frequently in people exposed to fungicides (OR 2.75, 95% CI 1.12–6.75) (19).

Pesticides may be linked to the development of PD through the direct effect of chemical substances on the nervous system and the dysregulation of the gut microbiome (2). Different classes of pesticides have direct mechanisms of action causing neurodegeneration, including oxidative stress, mitochondrial dysfunction, dopamine neurotoxicity, ubiquitin-proteasome system disruption, alpha-synuclein aggregation, and neuroinflammation (2, 20). The impact of pesticides on the gut microbiome occurs through impairing the gut barrier function and altering the microbiota diversity, which leads to modification of the availability and metabolism of the same pesticides caused by gut dysbiosis (2).

The study had many limitations. The total number of participants, mainly healthy controls, in the analysis was small. For example, we performed a post hoc power analysis for our logistic regression models (association between pesticide exposure and risk of developing PD) based on data from a previous study (9), and the estimated power of the present analyses was 15.2% for a total sample size of 270 participants. Thus, our models were underpowered for these analyses and the results may not be reliable for a small sample size.

Also, the environmental questionnaire (PDRFQ) used in the FOUND study has some issues: (I) the category with high pesticide exposure (maximum frequency: more than six times/year) impaired identifying a subgroup with participants with higher use [such as used in the LARGE-PD study—more than 30 times/year (9)]; (II) potential memory bias for lifelong pesticide exposure; (III) no data about the chemical group of active ingredients.

As strong points, the longitudinal design of the PPMI allowed an analysis of the effect of household pesticides on the progression of PD. Also, most people with PD were in the early stage of the disease and drug-naïve at baseline, resulting in a more homogenous sample. Considering the sample of participants in the PPMI cohort may increase in the following years, these data may be reanalyzed further.

In conclusion, PD was not associated with high household pesticide exposure in the PPMI study cohort. Participants with high fungicide exposure had a faster rate of cognitive dysfunction. The data suggest that environmental factors may also impact the clinical progression of PD. In the future, a higher sample size in the PPMI cohort and a longer period of follow-up of participants will enable the analysis of neuropsychological, imaging and CSF biomarkers to confirm the impact of fungicide exposure on cognition.

## Data availability statement

The original contributions presented in the study are included in the article/Supplementary material, further inquiries can be directed to the corresponding author.

## Ethics statement

The studies involving humans were approved by the Parkinson's Progression Markers Initiative cohort local Ethics Committees of the participating sites (Supplementary Table 2). The studies were conducted in accordance with the local legislation and institutional requirements. The participants provided their written informed consent to participate in this study.

## Author contributions

BS-L: Conceptualization, Data curation, Methodology, Writing – original draft, Writing – review & editing. AS: Writing – original draft, Writing – review & editing.
