# Supplementary material for: Exposure to household pesticides and Parkinson's disease in the Parkinson's Progression Markers Initiative cohort
Source: Front Neurol. 2024 Aug 12;15:1411468. doi: 10.3389/fneur.2024.1411468 (PMC11346339; doi:10.3389/fneur.2024.1411468)
Supplement: Supplementary file 1 [file Table_1.docx]

**Supplementary Table 1.** Methodological characteristics of studies which explored the association of household pesticide exposure and Parkinson’s disease

| **Author, Year** | **Country** | **Study design** | **Sample size Controls** | **Sample size PD** | **Frequency of pesticide use** | **Cumulative pesticide exposure** | **Categories of cumulative exposure** | | **High-tier exposure definition** | **Categories of pesticides** | **Statistic method** | **Covariants** |
| --- | --- | --- | --- | --- | --- | --- | --- | --- | --- | --- | --- | --- |
| Firestone, 2005 | USA | Population-based | 388 | 250 | Number of exposed days per year | Yes; Frequency x Duration | | Low; Medium; High | Not described | Insecticides, herbicides, fungicides, organophosphates | Logistic regression | Age, sex, smoking |
| Narayan, 2013 | USA | Population-based | 807 | 357 | Non/Rare (< 1x/year); occasional (2-11x/year); regular (=> 1x/month or more) | Yes; Frequency x Duration | | Never/rare use; Frequent use | Average frequency at or above the median exposure of exposed control | Organophosphates; Organothiophosphates | Logistic regression | Age, sex, smoking, race, education, family history of PD |
| Moura, 2023 | Brazil | Clinic-based | 374 | 562 | 1-5x/year; 6-10x/year; 11-30x/year; more than 30x/year | Not calculated | | Not high; High | More than 30x/year any time in lifetime | Not described | Logistic regression | Age, sex, smoking, geographic origin |
| Present study, 2024 | USA | Clinic-based | 64 | 206 | Rarely (1-2x/year); Occasionally (3-6x/year); Often (more than 6x/year) | Yes; Summed scores for lifetime, with log- and z-transformation | | Not high; High | Z-score of transformed lifetime summed score higher than 1 standard deviation | Insecticides, herbicides, fungicides | Logistic regression | Age, sex, smoking, origin, education |
